# Supplementary material for: Reaching a cell monolayer at the end of hiPSC differentiation enhances neural crest lineage commitment
Source: PLoS One. 2025 Sep 4;20(9):e0331046. doi: 10.1371/journal.pone.0331046 (PMC12410764; doi:10.1371/journal.pone.0331046)
Supplement: S1 Table — (DOCX) [file pone.0331046.s001.docx]

**Table S1:** Sequence of the primers used in gene expression analysis

| **Genetic Marker** | **Forward primer sequence** | **Reverse primer sequence** |
| --- | --- | --- |
| *OCT3/4* | GACAGGGGGAGGGGAGGAGCTAGG | CTTCCCTCCAACCAGTTGCCCCAAAC |
| *SOX10* | CTCTGGAGGCTGCTGAA | TGGGCTGGTACTTGTAGTC |
| *SNAI2* | CAGACCCTGGTTGCTTCAAG | GAGCCCTCAGATTTGACCTG |
| *PAX6* | GGCAACCTACGCAAGATGGC | TGAGGGCTGTGTCTGTTCGG |
| *SOX1* | GTGACATCTGCCCCCATC | GAGGCCAGTCTGGTGTCAG |
| *NANOG* | TGCCTGTGATTTGTGGGCCT | TTGCCTTTGGGACTGGTGGA |
