# Supplementary figures and images for: Reaching a cell monolayer at the end of hiPSC differentiation enhances neural crest lineage commitment
Source: PLoS One. 2025 Sep 4;20(9):e0331046. doi: 10.1371/journal.pone.0331046 (PMC12410764; doi:10.1371/journal.pone.0331046)

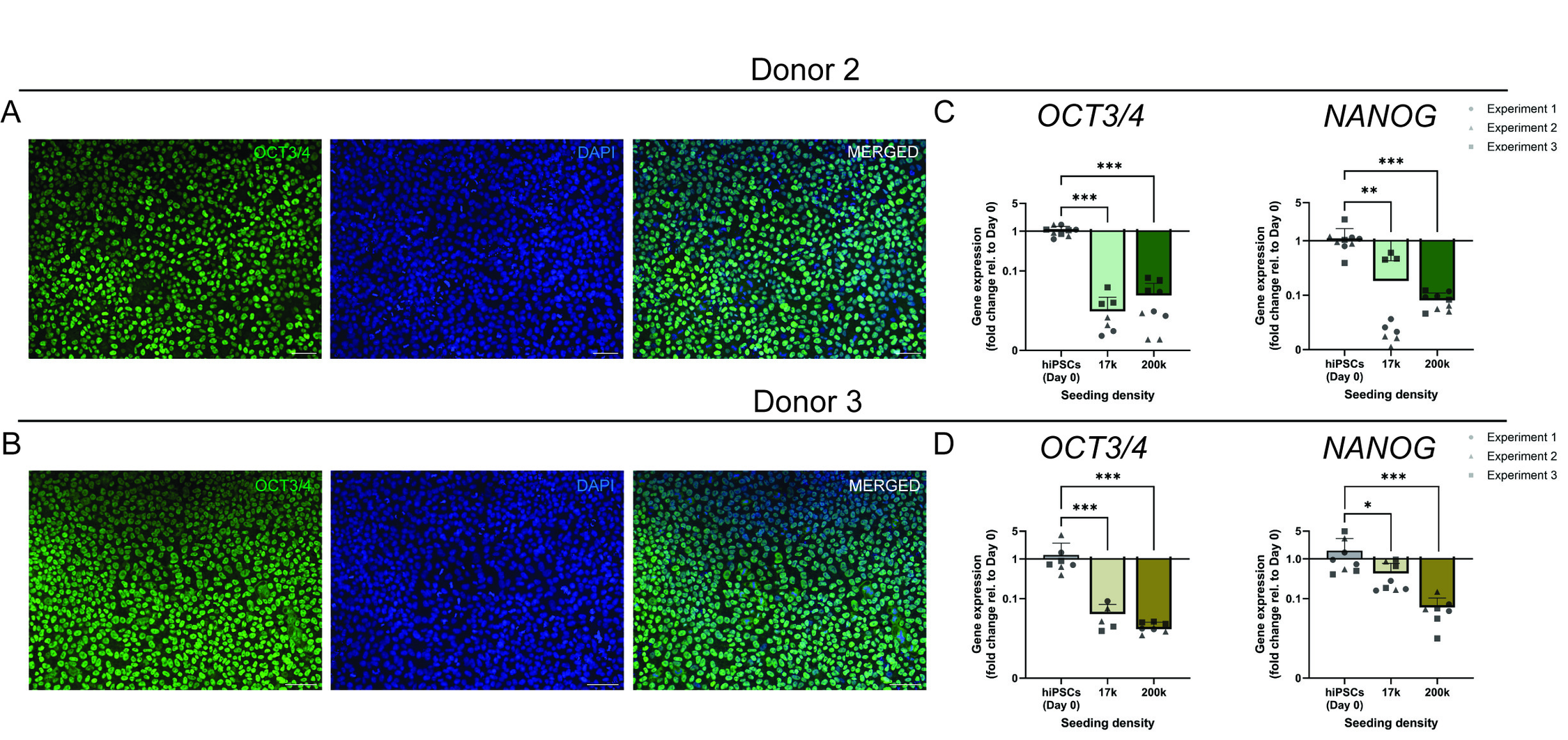

Supplement: S1 Fig — Immunocytochemistry analysis of OCT3/4 expression in PBMC-derived hiPSCs from donor 2 (A) and donor 3 (B) confirms extensive stemness marker expression. Scale bars = 100 µm. Gene expression analysis of OCT3/4 and NANOG in donor 2 (C) and donor 3 (D), show loss of pluripotency after NC induction and was assessed on undifferentiated cells (Day 0, grey bars) and after 8 days of neural crest differentiation, with a 17,000 cells/cm2 and 200,000 cells/cm2 initial seeding density. Differentiated NCSCs from donor 2 and donor 3 are represented in green and brown, respectively. Fold changes were calculated relative to the expression levels in hiPSCs (at Day 0). Error bars represent standard deviation (n = 3). *: p-value < 0.05, **: p-value < 0.01, ***: p-value < 0.001, ns: not significant. (TIF) [file pone.0331046.s002.tif]
